# Ultrasound - guided access during percutaneous nephrolithotomy: entering desired calyx with appropriate entry site and angle

**DOI:** 10.1590/S1677-5538.IBJU.2015.0622

**Published:** 2016

**Authors:** Abbas Basiri, Amir H. Kashi, Mehdi Zeinali, Mahmoodreza Nasiri, Reza Sarhangnejad, Reza Valipour

**Affiliations:** 1 Urology and Nephrology Research Center, Shahid Labbafinejad Medical Center, Shahid Beheshti University of Medical Sciences, Tehran, Iran; 2 Hasheminejad Kidney Center, Iran University of Medical Sciences, Tehran, Iran

**Keywords:** Ultrasonography, Nephrostomy, Percutaneous

## Abstract

**Objectives:**

To evaluate the success of ultrasonography directed renal access in entering the target calyx from proper entry site and in the direction of renal pelvis during percutaneous nephrolithotomy (PCNL).

**Materials and Methods:**

PCNL cases who were operated on by one fellow from May-June 2014 were included in this study. A vertically placed ultrasound probe on the patient flank in prone position was used to identify the preselected target calyx. Needle was advanced through needle holder and fluoroscopy was used to document the entered calyx, site and angle of entry.

**Results:**

Successful entering to the target calyx was achieved in 43 cases (91%). Successful entry with appropriate entry site and angle was observed in 34 cases (72%). Reasons for failure were minimal hydronephrosis, upper pole access and high lying kidneys.

**Conclusions:**

Although it is feasible to access a preselected calyx by ultrasonography guidance during PCNL, but entry to the calyx from the appropriate site and direction is another problem and needs more experience. In cases of minimal hydronephrosis, superior pole access or high lying kidneys, ultrasonography is less successful and should be used with care.

## INTRODUCTION

Percutaneous nephrolithotomy (PCNL) is now the treatment of choice for surgical management of large renal stones. Fluoroscopy has traditionally been the method for obtaining access to the pelvicalyceal system. Later ultrasonography access to the pelvicalyceal system has been introduced and popularised by some researchers (1–8). It has previously been shown that ultrasonography guided PCNL is equivalent to or even sometimes better than fluoroscopy guided PCNL in terms of stone free rate (1, 5, 9), operation duration (5), bleeding (5, 9) and complications (5).

A perfect percutaneous access tract to the pelvicalyceal system should be made through the tip of renal papilla in the targeted calyx and to be along the axis of renal calyx (10) so that the guide wire is passed into the pelvis and/or ureter. In fluoroscopy guided PCNL, entry to the targeted calyx is evident by 2 directional fluoroscopy images. There is a concern with ultrasonography guided PCNL for entry into the previously selected calyx especially for urologists who are not familiar with ultrasonography. We could not find any publication evaluating the precision of ultrasonography in targeting the desired calyx in the desired direction and through renal papillae.

The aim of this study was to access the effectiveness of ultrasonography guided PCNL renal access in entering the targeted calyx and in the direction of renal pelvis.

## MATERIALS AND METHODS

**Figure 1:**
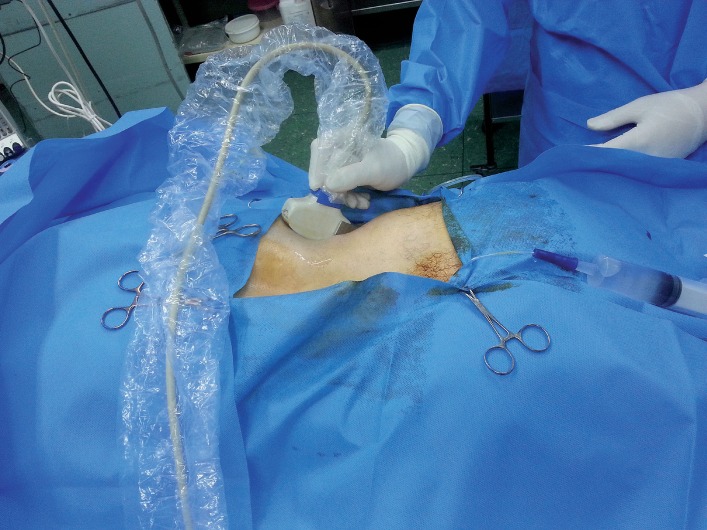
Ultrasonography probe was hold on the back of the patient parallel to the long axis of the kidney.

**Figure 2:**
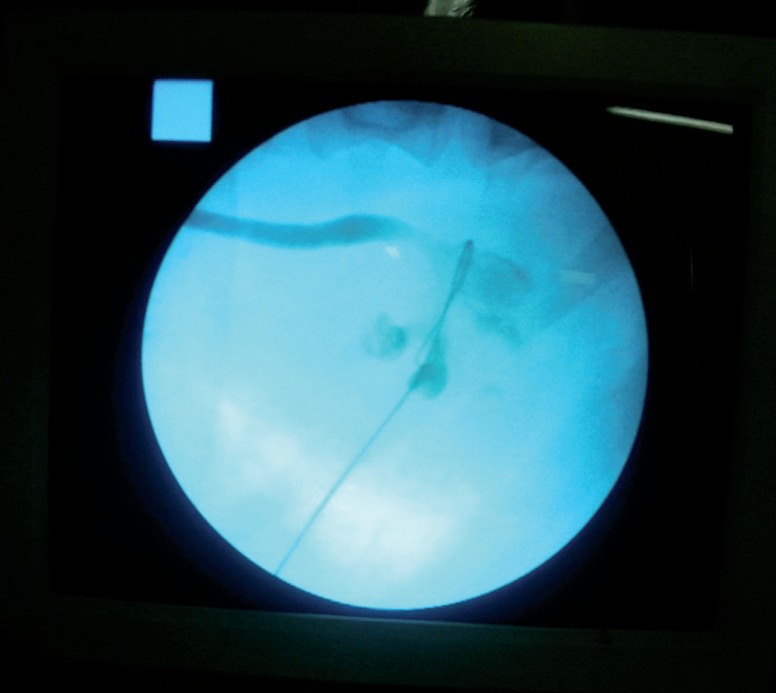
Documentation of guide wire entry site and angle by fluoroscopy after injecting contrast media from ureteral catheter. In this case guide wire was passed from proper entry site and coiled in renal pelvis

Patients who were candidate for PCNL in Labbafinejad Hospital and were operated by one fellow during May-June 2014 were included. PCNL cases included patients with large renal stones (>2cm) or patients with renal stones >1.5cm in horseshoe kidneys. No specific exclusion criteria were applied and typical cases for PCNL within the study period were included. The operating fellow had prior experience in fluoroscopy guided PCNL (>400 cases) and in ultrasonography guided PCNL (>50 cases). PCNL was done according to the standard protocol described before (3). In brief, cystoscopy was done in lithotomy position and a 4–6F ureteral catheter was inserted. Then the patient was turned into prone position. Ultrasonography was done by Siui Apogee 3800 (Guangdong, China) ultrasonography machine. A convex abdominal probe was placed on the flank of the patient parallel to the long axis of the kidney so that the 2D view included all possible calices from superior to inferior poles (Figure-1). The calyx which had been selected for access based on preoperative imaging (IVP or CT scan) was identified by ultrasonography. Then an 18Y gauge Shiba needle was advanced through a needle holder attached to the side of the ultrasonography probe. Entering the target calyx was confirmed by viewing the needle path on the ultrasonography monitor. Then the needle obturator was removed and saline was infused through the ureteral catheter. The outflow of urine from the needle proved the presence of the needle in the pelvicalyceal system. The guide wire was passed through the needle into the PC system until resistance was felt. Then radio-opaque contrast was injected through ureteral catheter and after opacifying the pelvicalyceal system, needle path and guide wire route were documented by fluoroscopy (Figure-2). If the entered calyx was different or not appropriate for PCNL or the calyx was entered from positions other than the papillae, the needle and guide wire were withdrawn and access was obtained under fluoroscopy guidance. The use of fluoroscopy here was as to confirm the accuracy of ultrasonography guided access and not as an adjuvant to ultrasonography guided access. In patients whom the obtained access was judged wrong and not feasible for safe PCNL by fluoroscopic images, a second access was obtained by means of fluoroscopy and the ultrasonography taken access was considered as failed.

**Figure 3:**
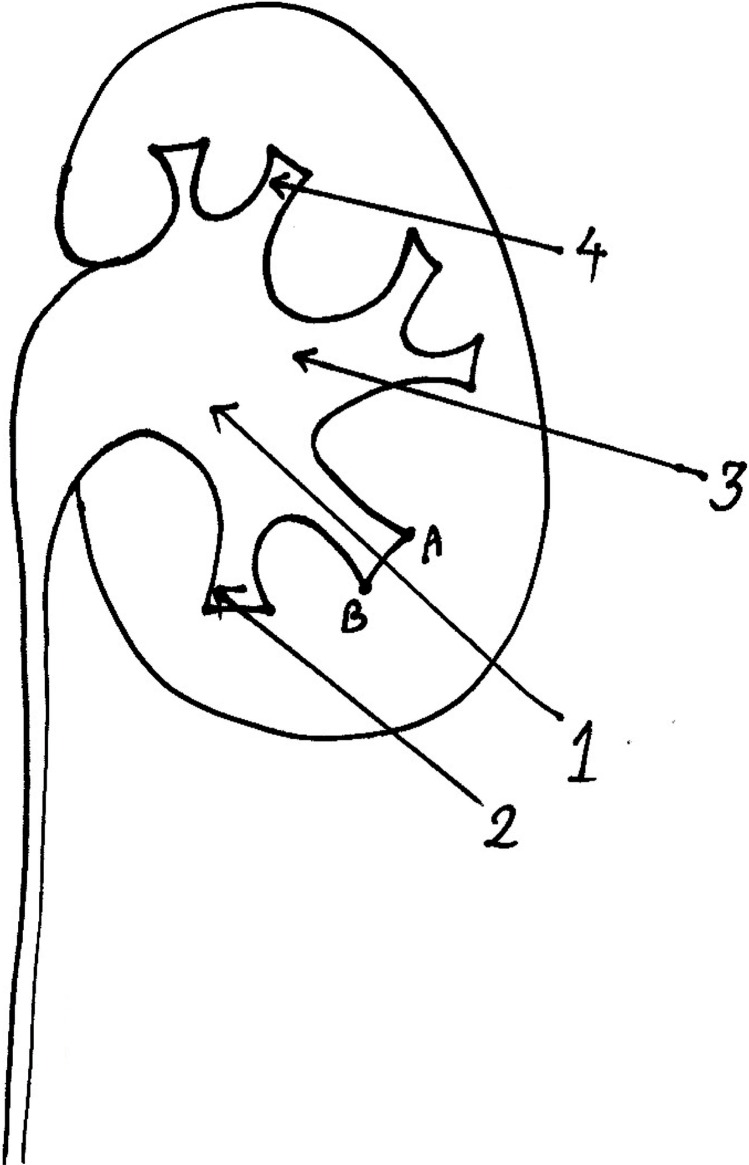
Definition of proper entry site and angle in this study. proper entry site was defined as the surface of renal papillae (from point A to point B). proper entry angle was defined as an angle that leads to renal pelvis (parallel to the axis of entered renal calyx).

In this study proper entry site into renal calyx was defined as entry from the surface of renal papillae and not from the lateral walls of the calyx or any other entry sites. Proper entry direction was defined as the direction or angle that leads to renal pelvis (Figure-3). Tract was dilated by guidance of fluoroscopy/ultrasonography and PCNL was performed by pneumatic lithoclast and stone fragments were removed by forceps.

Placement of nephrostomy or removal of ureteral catheter was done according to the judgement of the attending surgeons.

The ethics of this study was approved by the local committee institutional board review. All patients were informed about the study and informed consent obtained.

## RESULTS

52 patients were enrolled during the study period. 5 patients were excluded from the study and their data is not presented in the below sections. Reasons for exclusion from the study were: fat body resulting in poor ultrasonic view of the kidney (1 patient), ectopic kidney with poor ultrasonic view (1 patient), high lying kidneys with poor longitudinal ultrasonic view because of rib shadows or stones in superior calices making ultrasonic view of the stone by ultrasonic probe in longitudinal view difficult (3 cases).

Details of the 47 remaining patients and their operations have been outlined in Table-1. As explained in the methods section, access phase was totally guided by ultrasonography in all patients. Tract dilation was ultrasonography guided in 7 patients and with fluoroscopy guidance in 40 patients. No case of bowel injury or solid organ injury was observed in the studied patients. Successful entry into targeted calyx was observed in 43 cases (91%). Successful entry, proper entry site and appropriate direction (all) were observed in 34 cases (72%). In cases with proper entered calyx (n=43), appropriate entry site and coiling of guide wire were observed in 38 and 39 cases (88% and 91% respectively).

**Table 1:** Patients' demographic data and operations' characteristics.

Variable*	
Age, years	46.9±12.6
Gender, Male/Female	34/13
Positive Hx of PCNL	17 (36)
Positive Hx of OSS	6 (13)
Side, Left/Right	30/17
Access, L/Mid/U	25/20/2
Hydronephrosis, N/Mod/S	23/17/7
Stone location, U/Mid/L/P/Mul/Stag	2/2/12/13/10/8
Operation duration, minutes	55.3±21.7
Transfusion	1 (2)
Stone free rate	41 (87)

**hx** = History; **pcnL** = Percutaneous nephrolithotomy; **Oss** = Open stone surgery; **L** = Lower pole; Mid = Middle pole; **u** = Upper pole; **n** = Nil-minimal; **Mod** = Moderate; **s** = Severe; **p** = Pelvis; **Mul** = Multiple stone locations; **stag** = Staghorn

* Data is presented as mean±SD, N(%) or N/N

Table-2 compares the factors affecting false entered calyx. The percent of falsely entered calices increases steadily from 4% to 50% when the accessed calices changes from lower calices to upper calices (Table-2). False entered calices were observed in 17% of patients with nil-minimal hydronephrosis compared to 0% in patients with moderate or severe hydronephrosis. If hydronephrosis is recategorized into nil-minimal versus moderate-severe (2 categories) then the observed differences are statistically significant (fisher p-value=0.049). Age, side of operation, gender, history of open stone surgery or PCNL and stone location were not associated with false entered calices.

**Table 2:** The association of false entered calyx with studied variables.

Factors†		Wrong entered calyx		P-Value
		Yes	No	
Age, years		54.7±10.3	46.0±12.2	0.17
Access	Lower pole	1(4)	24(96)	0.10*
	Middle pole	2(10)	18(90)	
	Upper pole	1(50)	1(50)	
Side	Right	2(12)	15(88)	0.61
	Left	2(7)	28(93)	
Hydronephrosis	Nil-Minimal	4(17)	19(83)	0.06*
	Moderate	0(0)	17(100)	
	Severe	0(0)	7(100)	
Gender	Male	2(6)	32(94)	0.30
	Female	2(15)	11(85)	
Hx of OSS	Yes	1(17)	5(83)	0.43
	No	3(7)	38(93)	
Hx of PCNL	Yes	1(6)	16(94)	1.0
	No	3(10)	27(90)	
Stone location	Upper pole	1(50)	1(50)	0.14
	Middle pole	0(0)	2(100)	
	Lower pole	0(0)	12(100)	
	Pelvis	1(8)	12(92)	
	Multiple sites	2(20)	8(80)	
	Staghorn	0(0)	8(100)	

**Oss** = Open stone surgery; **pcnL** = Percutaneous nephrolithotomy

* Chi square p-value for linear by linear association

† Data are presented as mean±SD or N (%)

Table-3 compares factors influencing proper entry site and coiling of calyx in patients in whom the entered calyx was right (N=43). False entry site was observed in the only patient with upper pole access to the upper pole stone. (We excluded 3 patients with upper-middle pole stones in whom rib shadows precluded good longitudinal view of the kidney by ultrasonography probe). The percent of false entry site constantly decreased from 16% in nil-minimal hydronephrosis to 0% in severe hydronephrosis, however this difference was not statistically significant. False entry site was more often observed on the right side PCNLs (p=0.04). Failure of the guide wire to coil in calyx/pelvis was not associated with any studied variables. Failure to coil was observed in 3 patients (16%) with nil-minimal hydronephrosis, no case with moderate hydronephrosis and 1 case with severe hydronephrosis (Table-3). The patient with severe hydronephrosis in whom the guide wire failed to coil was a 53 years old male with staghorn left kidney stone that occupied the whole pelvis and most renal calices leaving little free space for guide wire to coil or move freely in the calyx.

**Table 3:** The associations of false entry site or failure of guide wire to coil with studied variables.

Factors†		False entry site		P-value	Failure to coil		P-value
		Yes	No		Yes	No	
Age, years		45.4±13.6	46.1±12.2	0.90	55.5±2.4	45.0±12.4	0.10
Access	Upper pole	1(100)	0(0)	0.12	0(0)	1(100)	0.12
	Middle pole	2(11)	16(89)		2(11)	16(89)	
	Lower pole	2(8)	22(92)		2(8)	22(92)	
Side	Right	4(27)	11(73)	0.04	3(20)	12(80)	
	Left	1(4)	27(96)		1(4)	27(96)	0.11
Hydronephrosis	Nil-Minimal	3(16)	16(84)	0.83	3(16)	16(84)	0.26
	Moderate	2(12)	15(88)		0(0)	17(100)	
	Severe	0(0)	7(100)		1(14)	6(86)	
Gender	Male	4(12)	28(88)	1.0	3(9)	29(91)	1.0
	Female	1(9)	10(91)		1(9)	10(91)	
Hx of OSS	Yes	2(40)	3(60)	0.09	0(0)	5(100)	1.0
	No	3(8)	35(92)		4(11)	34(89)	
Hx of PCNL	Yes	3(19)	13(81)	0.34	2(12)	14(88)	0.62
	No	2(7)	25(93)		2(7)	25(93)	
Stone location	Upper pole	1(100)	0(0)	0.29	0(0)	1(100)	0.55
	Middle pole	0(0)	2(100)		0(0)	2(100)	
	Lower pole	1(8)	11(92)		1(8)	11(92)	
	Pelvis	2(17)	10(83)		0(0)	12(100)	
	Multiple*	1(12)	7(88)		1(12)	7(88)	
	Staghorn	0(0)	8(100)		2(25)	6(75)	

**Oss** = Open stone surgery; **pcnL** = Percutaneous nephrolithotomy

* Multiple stone locations

† Data are presented as mean±SD or N(%)

## DISCUSSION

A perfect access for PCNL should pass from the papillae into the collecting system and direction of the needle path should be coincident with the long axis of the target calyx (11). There have been reports about the safety and success of ultrasonography directed renal access or solo-sono PCNL (1–6, 12). Nevertheless, we could neither find any article describing the success of ultrasonography in targeting the access to the pre-selected calyx nor we could find publications describing the situations regarding renal anatomy or stone morphology that make ultrasonography directed renal PCNL difficult or bothersome.

In this study, we observed a high success rate for entering targeted calyx by ultrasonography guided renal access. Reasons for failure were: 1) minimal/no hydronephrosis in the target calyx. The absence of hydronephrosis makes identification of the stone and its surrounding calyx difficult on ultrasonography especially by less experienced urology surgeons. We usually inject normal saline through ureteral catheter in patients with nil-minimal hydronephrosis to augment the magnitude of hydronephrosis during the access phase of the operation. Another alternative can be injection of diuretic agents before the access phase so that the access time is roughly in the peak action duration of the diuretic agent 2). Failure to enter the preselected calyx was less often observed in lower calices in comparison with middle to upper calices. Therefore, in difficult or borderline cases, selection of lower calyx for entry especially in equivocal cases may be associated with a higher success rate. As illustrated in the methods section, appropriate entry site in this study was defined as the convex area of the tip of the calyx and not its lateral walls (Figure-3). Reasons for false entry site were: 1) Stones in superior calices. To avoid shadows of ribs, we placed the ultrasonography probe beneath the rib cage. As a result, the angle of the needle, its path length and the visibility of the stone were more difficult when the stone was in the superior calices. Also, the path of the needle is from near the inferior pole of the kidney toward its superior pole. Then it seems imperative that the entry to the superior calyx will be from its lateral walls not from the convex surface of its papillae (Figure-3). To overcome such a problem, it is possible to place the ultrasonography probe transversely on the patient flank instead of placing it vertically and perform the access beneath the ribs or even from the window between the ribs. This was not included in the study protocol and it is possible that the identification of the target calyx in relation to other calices would be more difficult on a transverse plan. Our personal experience is that it is possible to perform intercostal access by ultrasonography guidance when ultrasonography probe is placed transversely between ribs. In this method, the target calyx can be selected by cephalic-caudal movement of ultrasonography probe. However, more experience is required due to rib shadows and narrow entry space on skin 2). High lying kidneys. The same points and problems described for superior pole stones are also pertinent to high lying kidneys. After experiencing false entry site in the patient with superior pole stone, we excluded high lying kidneys when the ultrasonography view of the whole kidney was not possible because of rib shadows.

In univariate analysis, right side PCNLs were associated with more frequent false entry sites (Table-3). After performing a multivariable logistic regression analysis, the association of laterality was no longer statistically significant when hydronephrosis and accessed calyx were included in the equation.

In order to use ultrasonography for taking access in PCNL, there should be a clear view of the kidney calices on ultrasonography monitor. In situations in which this image is not clear, the success of ultrasonography in properly targeting the access calyx, its entry site or entry angle can be impaired. In our limited experience, this scenario happened in the following situations 1). A very fat patient with normal lying kidney with a 2cm stone in the lower calyx, 2) One muscular patient with body mass index of 27 with low lying mal-rotated kidney with a pelvis stone, 3) Three patients with stones in middle or upper kidney calices so that when the ultrasonography probe was placed longitudinally beneath the ribs, the view of the whole kidney or the segment containing the stone was not clear. In a few cases we tried to overcome this problem by angling the ultrasonography probe end caudally and pressing it on the patient back below the ribs but in these cases, this simple manoeuver could not solve the problem. Nevertheless, in such cases it is possible to obtain access to lower calyx without the need to observe the calices containing stones on ultrasonography monitor and then perform PCNL. In the protocol of the current study, we requested to have the image of kidney calices and containing stones which was not possible in the above cases but just for the sake of performing PCNL, it is enough to obtain ultrasonography guided access through a lower calyx and then perform PCNL by rigid or flexible nephroscope. Having mentioned these solutions, we think it is still safer to have fluoroscopy equipment at hand in such cases in case of failed ultrasonography guided access.

We do acknowledge the operator dependency of ultrasonography a limitation of the results of this study. The experience of the operator can influence the obtained results. Nevertheless, there has been a publication by Japtag et al. in which the guidance of access by fluoroscopy versus ultrasonography yielded similar outcomes in hands of a trainee urologist with little prior experience in PCNL (13). It is necessary here to emphasize that fluoroscopy was not used in this study as an adjuvant to ultrasonography for accessing the kidney but ONLY to verify the access taken by the surgeon. In cases that the access was judged wrong by fluoroscopic images, a second access was taken by means of fluoroscopy and the ultrasonography access was considered as failed and analysed accordingly.

## CONCLUSIONS

Although it is feasible to access a preselected calyx by ultrasonography guidance during PCNL, but entry to the calyx from the appropriate site and direction is another problem and needs more experience. In patients with minimal hydronephrosis, upper pole access or high lying kidneys this success rate is lower or the entry site into the calyx is less perfect. In such cases, it is advisable to have fluoroscopy equipment at hand.
